# Autism Spectrum Disorder Associated With a CACNA1I Variant of Uncertain Significance: A Case Report

**DOI:** 10.7759/cureus.102980

**Published:** 2026-02-04

**Authors:** Mohammed A Al Medawi, Majed M Alshehri, Mohammed Saeed ALmasodi Asiri, Sarah Alsubaie

**Affiliations:** 1 Psychiatry, Armed Forces Hospital - Southern Region, Khamis Mushait, SAU; 2 Psychiatry, Mental Health Hospital, Abha, SAU; 3 Psychology, Armed Forces Hospital - Southern Region, Khamis Mushait, SAU

**Keywords:** autism spectrum disorder, cacna1i, genotype–phenotype correlation, t-type calcium channel, variant of uncertain significance (vus)

## Abstract

Autism spectrum disorder (ASD) is a highly heritable neurodevelopmental condition with multifactorial etiologies, and accumulating evidence suggests that ion channel dysfunction may contribute to a subset of phenotypes. We report a six-year-old Saudi girl with early-onset and persistent deficits in social communication, profound speech impairment, stereotyped behaviors, sensory dysregulation, and significant adaptive and cognitive delays. Standardized assessments demonstrated moderate global delay (Vineland composite score 42), clinically significant social communication impairment (SRS-2 total T-score 64, with earlier documentation exceeding 90), and moderate nonverbal cognitive delay (Leiter-3 nonverbal IQ 43). Whole-exome sequencing (CentoGenome® MOX 1.0 Solo) identified a heterozygous CACNA1I missense variant (NM_021096.3:c.6028G>T; p.Ala2010Ser) classified as a variant of uncertain significance, not present in population databases and interpreted as potentially de novo, with a possible splice effect flagged despite largely benign in silico predictions. The child received multidisciplinary interventions, including ABA-based behavioral therapy, speech therapy, occupational therapy, and risperidone for behavioral dysregulation, with mild improvement in social responsiveness but persistent minimal language and limited adaptive functioning. This case adds to emerging reports linking CACNA1I variation to neurodevelopmental phenotypes characterized by prominent speech impairment and highlights the clinical challenge of counseling families when a potentially relevant de novo finding remains classified as a variant of uncertain significance.

## Introduction

Autism spectrum disorder (ASD) is defined as a neurodevelopmental condition characterized by persistent deficits in social communication and social interaction, alongside restricted, repetitive patterns of behavior, interests, or activities, with onset in the early developmental period and causing clinically significant impairment in social, occupational, or other important areas of functioning. According to the Diagnostic and Statistical Manual of Mental Disorders, Fifth Edition, Text Revision (DSM-5-TR), diagnosis requires the presence of deficits across all domains of social communication and interaction, in addition to at least two types of restricted or repetitive behaviors, with symptoms not better explained by intellectual disability or global developmental delay. The DSM-5-TR further specifies ASD severity using three levels based on required support: Level 1 (requiring support), Level 2 (requiring substantial support), and Level 3 (requiring very substantial support), determined by the degree of impairment in social communication and the impact of restricted/repetitive behaviors on daily functioning. Similarly, the ICD-11 conceptualizes ASD as a spectrum disorder marked by impairments in reciprocal social interaction and communication, together with restricted, repetitive, and inflexible patterns of behavior, with symptom severity varying according to developmental level and environmental demands [1-3]. Genetic studies have implicated hundreds of risk loci, many encoding synaptic and ion channel proteins critical for neuronal excitability and plasticity [4]. Among these, the CACNA1I gene, located on chromosome 22q13.1, encodes the Cav3.3 T-type calcium channel that regulates thalamic oscillations, cortical excitability and sleep-wake transitions [5].

Mutations in CACNA1I have been linked to neurodevelopmental disorders with speech impairment with or without seizures (OMIM 620114) and neuropsychiatric phenotypes such as schizophrenia and bipolar disorder [6-9]. Latest reports connecting CACNA1I variants to ASD remain limited. Our contributes to the growing evidence that subtle channelopathies may participate in ASD pathophysiology even when the identified variants are classified as variants of uncertain significance (VUS) [5].

Why this case is notable

The variant p.(Ala2010Ser) is not reported in population databases and involves a residue in the canonical CACNA1I coding sequence. CentoGenome classified it as a class 3 variant (VUS) but noted a likely splice effect in research findings [10]. The patient shows severe speech impairment with minimal expressive vocabulary, early absent social communication and sustained stereotypies from infancy. Such an early, prominent speech phenotype aligns with neurodevelopmental disorder with speech impairment with or without seizures (NEDSIS) descriptions and with published CACNA1I cases, but each published case remains rare [6-8].

The dataset includes repeated, objective psychometrics (Vineland, Social Responsiveness Scale, Second Edition (SRS-2), Assessment of Basic Learning Abilities-Revised (ABLA-R), Sensory Profile 2), multidisciplinary notes and a detailed trio-style genetic report. This density supports phenotype-genotype correlation despite VUS classification.

The case underscores practical issues for clinicians: how to interpret a gene with growing evidence but limited definitive pathogenic calls and how to counsel families when the variant is de novo but classified as VUS [10].

## Case presentation

A six-year-old Saudi girl presented to the child and adolescent psychiatry clinic with global social and communication deficits. She is the only child of consanguineous parents and resides with both. The child lives with both parents. The caregiver report indicated a stable home environment and consistent caregiving. The mother described strong emotional attachment and active engagement in daily care; however, reciprocal social behaviors (eye contact, shared enjoyment, and social bids) were limited from infancy, contributing to caregiver stress.

Family history was negative for autism, intellectual disability, epilepsy, major psychiatric disorders, or known genetic syndromes. There was no reported family history of substance use disorders. No history of significant domestic violence or chronic family trauma was reported. There was no reported history of physical, emotional, or sexual trauma to the child.

Presenting complaint

Since infancy, her parents noted the absence of meaningful speech, lack of response to her name, poor eye contact and minimal social reciprocity. She exhibited repetitive movements and vocalisations, including humming and spinning. Her expressive vocabulary comprised fewer than ten words, with slow progress over recent months despite early interventions.

Perinatal and developmental history

Maternal age at delivery was 23 years, and paternal age was 24 years. The pregnancy was uneventful, culminating in a full-term vaginal delivery assisted by forceps. The newborn required a short NICU stay due to minor cranial bleeding but had no lasting neurological complications. Birth weight was 2000 g. Early motor milestones were delayed; she walked independently at one year without prior crawling. Before age two years, early vocalizations and babbling were present; however, the child did not develop consistent, meaningful single words or functional two-word phrases. Gestural communication (pointing, showing) was minimal. Caregivers reported regression around age 2 years, characterized by reduced babbling/vocalizations and loss of the few emerging word-like utterances, followed by persistent minimal expressive language despite intervention. Toilet training was not achieved, and fine motor coordination remained poor. Hearing was evaluated using otoacoustic emissions (OAE) and auditory brainstem response (ABR) testing, with results within normal limits, reducing concern for hearing impairment as a primary contributor to language delay.

Behavioural, communication and developmental profile

From early life, she displayed profound deficits in socialization and communication. She rarely responded to her name, avoided eye contact and did not initiate or maintain social interaction. Nonverbal communication, such as pointing, nodding or waving, was absent. She showed no sharing of interests or achievements with caregivers and engaged in no symbolic play. Repetitive behaviours included lining up toys, circling, humming and repeating phrases. Sensory behaviours involved mouthing, sniffing and selective eating and clothing preferences, though some tolerance improvement was reported. Episodes of aggression, tantrums and self-injurious behaviour were observed. Overall cognitive and adaptive functioning remained poor with only modest improvement under therapy. The child did not demonstrate age-appropriate peer relationships; the caregiver's report indicated minimal interest in other children and limited parallel or interactive play.

Mental state and psychiatric examination

At clinical, she appeared disengaged with minimal interest in people and an absent response to name. Affect was negative, and her participation was limited. Differential diagnoses included ASD Level 2 with possible intellectual disability and behaviour problems secondary to ASD.

Medical and treatment history

There were no seizures or significant dysmorphic features. At the time of the current presentation, the patient had already been receiving speech therapy, occupational therapy, psychological intervention, and special education services. Speech therapy had been ongoing for two years, with 45 minutes sessions 2 times/week, primarily targeting language development and functional communication; the family reported no improvement in vocabulary, following instructions. Occupational therapy had been ongoing for 18 months, with 45-minutes sessions once times/week, focusing on sensory regulation and daily living/fine-motor skills; with no improvement was noted in tolerance to sensory input and self-care. The patient was receiving psychological intervention in the form of ABA-based behavior modification for 12 months, with 60-minutes sessions three times/week, targeting behavior regulation and adaptive skills; partial improvement was observed in reduced tantrums.

Regarding education, the patient had been enrolled in a day-care center school/program for two years and received instruction/support from a special educator in a self-contained setting. Special education services had been provided for 2 years; overall academic and functional performance was described as below age level, with ongoing difficulties mainly in functional communication, attention, and peer interaction.

Pharmacological treatment with risperidone had also been initiated prior to the formal multidisciplinary diagnostic evaluation and was continued thereafter with ongoing monitoring. Risperidone therapy had been ongoing for approximately 12 months at the time of the most recent follow-up; treatment was initiated at a low dose and progressively titrated to 2 mg twice daily. Risperidone was initiated approximately 12 months prior to the most recent follow-up for management of behavioral dysregulation.

Psychological and cognitive assessments

On the Vineland Adaptive Behavior Scales, Third Edition (Vineland-3), the adaptive behavior composite (ABC) was 42 (low range), with domain scores as follows: Communication = 45 (low), Daily living skills = 40 (low), Socialization = 43 (low), consistent with moderate global delay. The Social Responsiveness Scale-Second Edition (SRS-2) indicated mild-to-moderate deficits in social communication and restricted behaviours with a total T-score of 64. Subscales showed social awareness within normal limits (T = 51), social cognition (T = 68) and social communication (T = 69) in the moderate range, and social motivation was normal (T = 52). Restricted interests/repetitive behaviour scored mild (T = 64). These findings reflected clinically significant impairments in reciprocal social functioning. Psychological evaluation revealed variable engagement, refusal behaviours and negative affect. An earlier behavioral rating (SRS-2 Total T-score >90) was documented at age five years by a psychologist. During assessments, she resisted participation and displayed temper tantrums.

Speech and language evaluation

Her informal speech-language assessment showed no functional verbal communication. Expressive and receptive language were seen to be delayed. She relied on pulling a caregiver’s hand or pointing to communicate needs. Babbling persisted with early language regression at age two. She had been enrolled in a private early intervention program providing structured behavioral and communication support for approximately 24 months before the current evaluation, and she was / was not still attending at the time of presentation.

Educational and cognitive testing

On the assessment of basic learning abilities (ABLA-R), she passed imitation, position discrimination and visual discrimination tasks (Levels 1-3) but failed higher-level tasks involving match-to-sample visual and auditory-visual discrimination (Levels 4-6). These findings suggested limitations in abstract reasoning and multimodal integration.

A nonverbal intelligence assessment was completed using the Leiter International Performance Scale, Third Edition (Leiter-3). The child was cooperative and completed all tasks without difficulty. Her performance showed marked cognitive delay. The cognitive composite (Nonverbal IQ) measured 43, which lies within the moderate delay range. Subtest analysis showed severe deficits in visual interference tasks (Figure Ground, scaled score 0) and in rule-based classification and analogical reasoning (Classification/Analogies, scaled score 1). Form completion was also weak, with a scaled score of 2, indicating poor deductive organization and difficulties completing fragmented patterns. Sequential order was relatively stronger with a scaled score of 7, placing it in the below-average range and suggesting better performance on tasks requiring pattern tracking and sequential reasoning. Taken together, these findings support moderate nonverbal cognitive delay consistent with her broader developmental profile (Table 1).

**Table 1:** Leiter-3 nonverbal IQ and subtest performance

Subtest	Raw Score	Scaled Score	Interpretation
Figure Ground	1	0	Moderate delay
Form Completion	6	2	Very low to mild delay
Classification/Analogies	6	1	Very low to mild delay
Sequential Order	13	7	Below average
Cognitive Composite (Nonverbal IQ)	—	—	IQ = 43 (Moderate delay)

Sensory profile and occupational

Therapy occupational assessment using the Sensory Profile 2 demonstrated marked abnormalities across multiple domains. Seeking behaviours were elevated (scores 48-60) as were sensitivity (43-53) and avoiding behaviours (47-59). Touch processing, attention responses and behavioural modulation scores were also elevated, indicating atypical sensory reactivity and associated emotional dysregulation.

Physical and neurological examination

Physical and neurological examinations were performed and revealed no focal neurological deficits or dysmorphic features. Vital signs were within normal limits for age with no significant findings.

Genetic and laboratory investigations

Whole-exome sequencing (CentoGenome® MOX 1.0 Solo, CENTOGENE) identified a heterozygous missense variant in CACNA1I (NM_021096.3:c.6028G>T; p.Ala2010Ser), which is classified as a Variant of Uncertain Significance (VUS, Class 3) [10]. This variant was not present in population databases (gnomAD, 1000G, CentoMD) and was interpreted as potentially de novo. In silico predictions were largely benign (PolyPhen: benign; SIFT: tolerated; MutationTaster: polymorphism), though a possible splice effect was flagged. Related disorder (OMIM 620114) involves neurodevelopmental delay and speech impairment with or without seizures [6-8].

Carrier findings included HBB (p.Glu7Val; rs334) consistent with heterozygous sickle-cell trait and TSPEAR (p.Glu596del; rs782084367), which is seen very rare in-frame deletion without phenotypic overlap. No ACMG secondary actionable variants were seen (Table 2) [10].

**Table 2:** CACNA1I, HBB and TSPEAR: Variant annotation, clinical classification and relevant notes.

Gene	cDNA	Protein Change	Zygosity	Type	Population Frequency	Classification	Notes
CACNA1I	NM_021096.3:c.6028G>T	p.(Ala2010Ser)	Heterozygous	Missense	Not found	VUS (Class 3)	De novo; potential splice effect; possible link to neurodevelopmental delay
HBB	NM_000518.4:c.20A>T	p.(Glu7Val) rs334	Heterozygous	Missense/SNP	Common	Likely pathogenic (carrier)	Carrier for hemoglobinopathy
TSPEAR	NM_144991.2:c.1788_1790delAGA	p.(Glu596del) rs782084367	Heterozygous	In-frame deletion	Very rare	Reported	No clinical overlap with phenotype

Diagnosis

The diagnosis of autism spectrum disorder, Level 2, was based on persistent deficits in social communication, restricted and repetitive behaviors, early developmental onset, and functional impairment across settings, supported by standardized assessments (SRS-2, Vineland-3) and multidisciplinary clinical evaluation. Moderate global developmental delay/intellectual disability (Vineland 42).

Genetic finding: heterozygous CACNA1I p.(Ala2010Ser) variant of uncertain significance, de novo, potentially related to the speech impairment phenotype [6-8]. Carrier status for HBB p.(Glu7Val) noted.

Diagnostic challenges

The main diagnostic challenge in this case was differentiating isolated language delay from autism spectrum disorder with global developmental delay in the context of significant sensory dysregulation and behavioural problems. Hearing impairment and primary intellectual disability were carefully evaluated and excluded through appropriate audiological and cognitive assessments. The interpretation of the heterozygous CACNA1I variant as a VUS also posed a challenge for genetic counselling, as the variant may contribute to the phenotype but cannot be considered definitively pathogenic [10]. No additional competing diagnoses or major uncertainties remained after the multidisciplinary assessment.

Following the current presentation, the audiological evaluation was reviewed and confirmed normal hearing sensitivity, supporting exclusion of hearing impairment as a contributor to speech delay.

Although moderate cognitive delay was identified, primary intellectual disability was considered less likely given the presence of core autistic features, early social-communication deficits, restricted behaviors, and disproportionate language impairment relative to nonverbal reasoning abilities. The multidisciplinary assessment involved a child psychiatrist, clinical psychologist, speech-language pathologist, occupational therapist, and special educator.

Patient’s caregiver perspective

The patient’s mother described the diagnostic and treatment journey as both challenging and reassuring. She reported that, for several years, she felt worried and confused by her daughter’s lack of speech, eye contact, and social interaction, and was concerned that she had done something wrong as a parent. Receiving a clear diagnosis of autism spectrum disorder, along with an explanation of the genetic findings, helped her to understand her child’s difficulties and to adjust her expectations. She emphasized that the structured interventions and ongoing support from the multidisciplinary team have made daily life more manageable and have given the family hope that her daughter will continue to make gradual progress, even if improvements are slow.

Management and current status

The patient was enrolled in a comprehensive multimodal intervention program beginning shortly after diagnosis. Behavioural therapy based on applied behaviour analysis (ABA) principles was initiated at 10-12 hours per week, focusing on enhancing communication, reducing self-injurious behaviour, and improving adaptive functioning. These sessions have been delivered consistently for approximately 14 months.

Speech and language therapy was provided twice weekly, each session lasting 45-60 minutes, with emphasis on receptive and expressive language skills, early social-pragmatic abilities, and functional communication strategies. Occupational therapy sessions were conducted once weekly for 60 minutes, targeting fine motor coordination, sensory modulation, and daily living skills. Both therapies have continued regularly for 12-14 months with good family adherence.

Pharmacological treatment with risperidone was started at 0.25 mg nightly for behavioural dysregulation, including aggression and severe tantrums. The dose was gradually titrated over 3-4 weeks to a maintenance dose of 2 mg twice daily, with regular clinical monitoring. The patient has remained on this regimen for 12 months, with adherence assessed by caregiver report and pharmacy refill monitoring. Tolerability was evaluated at each follow-up visit, including review of appetite, weight, sleep, extrapyramidal symptoms, and daytime sedation.

Over the follow-up period, the patient demonstrated mild improvement in social responsiveness and limited language gains were noted. The child remains largely non-verbal, untrained in toileting and continues to show sensory hypersensitivity and limited adaptive functioning. Genetic counselling and further segregation testing were recommended to clarify the clinical significance of the CACNA1I variant [10]. Ongoing psychoeducation for the caregiver and multidisciplinary support remain integral components of care. The total clinical follow-up period was approximately 12-14 months.

According to adverse events

No adverse or unanticipated events related to pharmacological or non-pharmacological interventions were observed during the treatment period or follow-up.

Structured timeline

Key clinical events, assessments, and interventions are summarized in Table 3.

**Table 3:** Timeline of key clinical events, assessments and interventions

Age / Time	Clinical Events, Assessments, and Interventions
Birth	Full-term vaginal delivery with forceps; brief NICU stay for minor cranial bleeding; no lasting neurological complications.
0–2 years	Early social-communication deficits: poor eye contact, limited response to name, minimal babbling, repetitive humming/spinning.
Around 2 years	Regression of early vocalizations; persistent minimal language output; emerging repetitive behaviours and sensory dysregulation.
2–4 years	Absence of symbolic play; delays in fine motor and adaptive functioning; no toilet training achieved; increasing parental concern.
4–5 years	Initial developmental/psychological assessments: Vineland indicated moderate global delay; behavioural issues (tantrums, aggression, self-injury) documented.
5–6 years	Comprehensive multidisciplinary evaluation (SRS-2, ABLA-R, Leiter-3, Sensory Profile 2). Diagnosis: ASD Level 2 + global developmental delay/intellectual disability.
6 years	Whole-exome sequencing performed (CentoGenome® MOX 1.0 Solo). Heterozygous CACNA1I p.(Ala2010Ser) variant of uncertain significance identified. Genetic counseling recommended.
6–7 years (follow-up)	Continued behavioural (ABA-based) therapy, speech therapy, occupational therapy. Risperidone initiated and titrated for behavioural dysregulation. Mild improvement in social responsiveness. Persistent minimal language, sensory issues, limited adaptive functioning.

## Discussion

The identification of a CACNA1I variant in this child provides a valuable opportunity to examine the contribution of calcium channel dysfunction to ASD phenotypes. CACNA1I encodes the Cav3.3 subunit, a T-type calcium channel predominantly expressed in the thalamus and prefrontal cortex. These channels regulate neuronal excitability, sleep spindles and sensory gating, functions often disrupted in autism and schizophrenia [5,9].

Previous studies linked rare CACNA1I variants to neurodevelopmental syndromes featuring speech delay or cognitive impairment and seizures, but ASD-specific associations remain rare and phenotypically diverse [6-8]. The present variant (p.Ala2010Ser) resides within a conserved intracellular domain important for channel inactivation kinetics. Although current in silico tools predict benignity, experimental models suggest that subtle structural alterations in Cav3.3 can modulate thalamocortical oscillations, thereby influencing social cognition and sensory processing [5].

The proband’s phenotype, mild autistic symptomatology, profound speech delay and sensory dysregulation without seizures mirrors previous CACNA1I-linked cases but lacks epileptiform manifestations. The absence of other pathogenic variants and negative secondary findings strengthens the potential genotype-phenotype relevance despite current VUS classification [10]. From a mechanistic standpoint, dysfunction of T-type calcium channels can alter neuronal rhythmicity, leading to impaired signal integration within cortical-subcortical networks [5]. In animal models, CACNA1I knockdown results in abnormal social behavior and hyperactivity, suggesting that channelopathies may contribute to ASD endophenotypes even in the absence of overt epileptic phenotypes [8].

Similar to previously reported CACNA1I-associated cases, this patient exhibited prominent speech impairment and neurodevelopmental delay; however, ASD presentations linked to CACNA1I remain rare and heterogeneous. Clinically, this case emphasizes the necessity for dynamic variant interpretation. The ACMG framework classifies variants based on population frequency, computational data and functional studies, yet many neurodevelopmental variants remain VUS due to limited functional characterization [10]. Longitudinal follow-up, family segregation testing and electrophysiological analysis are warranted to clarify the pathogenic contribution of such variants [10].

The child’s mild ASD profile on SRS-2 contrasts with earlier psychiatric documentation of more severe impairment (SRS > 90), illustrating developmental variability and therapeutic impact. Her gradual progress under multidisciplinary therapy highlights the benefit of early, structured intervention irrespective of genetic etiology.

Clinically, this case highlights the importance of cautious genetic counseling when potentially relevant variants remain classified as VUS. At a policy level, it underscores the need for improved access to functional genomic studies and longitudinal reclassification frameworks in neurodevelopmental disorders.

## Conclusions

Our case is important as it adds value to emerging evidence implicating CACNA1I variants in autism spectrum pathophysiology. Although classified as a VUS, the p.Ala2010Ser variant may contribute to neurobehavioral modulation through calcium channel dysregulation. We suggest that continuous re-evaluation of such variants and integration of electrophysiological data and larger cohort studies can help to delineate CACNA1I’s role in ASD. Clinicians should consider ion channel gene testing in idiopathic ASD with pronounced sensory and speech deficits.

Standard management of ASD includes early behavioral interventions, speech and occupational therapies, individualized educational support, and pharmacological treatment for associated behavioral symptoms when indicated. Early, sustained multidisciplinary intervention remains central to optimizing functional outcomes.
